# Association between smoking status and non-alcoholic fatty liver disease

**DOI:** 10.1371/journal.pone.0325305

**Published:** 2025-06-09

**Authors:** Hyun Joe, Jung-Eun Oh, Yong-Jin Cho, Hwang-Sik Shin

**Affiliations:** 1 Department of Family Medicine, College of Medicine, Soonchunhyang University College of Medicine, Soonchunhyang University Seoul Hospital, Seoul, Korea; 2 Department of Family Medicine, College of Medicine, Soonchunhyang University Cheonan Hospital, Cheonan-si, Chungnam, Republic of Korea; University of Diyala College of Medicine, IRAQ

## Abstract

**Background:**

The relationship between cigarette smoking and nonalcoholic fatty liver disease (NAFLD) remains controversial. Recent studies have demonstrated that cigarette smoking is a significant risk factor for the development of NAFLD. This study aimed to examine the association between smoking and NAFLD according to smoking status among Korean males, and to examine the relationship between smoking cessation and NAFLD.

**Methods:**

This cross-sectional study included data from 12,241 adult males who underwent health checkups at a university hospital health promotion center between January 2018 and December 2019. Fatty liver was diagnosed using abdominal ultrasonography. The participants were categorized according to self-reported smoking status, pack-years, and period of smoking cessation. Odds ratio (OR) and corresponding 95% confidence interval (CI) for NAFLD were calculated using logistic regression analysis.

**Results:**

After adjusting for confounding factors, the OR for NAFLD was 1.190 (95% CI 1.071–1.322, *P *= .001) among ex-smokers. Among current smokers, the risk for NAFLD increased with an increase in the amount of cigarette smoking (10–20 and ≥20 pack-years versus [vs.] never smoker, adjusted OR [aOR] 1.289 [95% CI 1.107–1.500]; *P* = .001 and 1.235 [95% CI 1.043–1.461]). The prevalence of NAFLD was inversely associated with the duration of smoking cessation (< 10 years vs. 10–20 years and ≥ 20 years; aOR 0.748 [95% CI 0.638–0.876], *P* < .001 and 0.750 [95% CI 0.592–0.950], *P* = .017, respectively).

**Conclusion:**

Cigarette smoking was significantly associated with increased odds of NAFLD, whereas smoking cessation for more than 10 years was associated with decreased odds.

## 1 Introduction

Non-alcoholic fatty liver disease (NAFLD) is characterized by the presence of hepatic steatosis affecting more than 5% of hepatocytes, as identified by imaging or histology, in the absence of secondary causes such as significant alcohol consumption, hepatotoxic drug use, viral hepatitis, and inherited liver disorders [1]. NAFLD includes nonalcoholic fatty liver, nonalcoholic steatohepatitis, and NAFLD-related cirrhosis [2].

NAFLD is a common chronic liver disease that affects approximately one-quarter of the world’s adult population, and its prevalence is projected to gradually increase [3]. In Korea, the diagnostic prevalence using abdominal ultrasonography in a meta-analysis was 32.9% [4]. Recent studies have reported that NAFLD was associated with insulin resistance, cardiovascular disease, diabetes, and metabolic syndrome [5–7].

In East Asia, the prevalence of NAFLD has been reported to be approximately 25–30% in China [8] and 20–25% in Japan [9], which is comparable to the prevalence in South Korea. These regional data help contextualize the public health burden of NAFLD and emphasize the need for further investigation into modifiable risk factors such as smoking.

Poor diet and lack of exercise have increased the prevalence of various metabolic diseases, such as metabolic syndrome, diabetes, and NAFLD, along with an increase in the obese population, and the proportion of NALFD in chronic liver disease is gradually increasing [10,11]. Recent studies have reported an increase in liver-related mortality with an increase in the prevalence of NAFLD [12,13]. Therefore, it is necessary to develop preventive strategies by identifying correctable risk factors associated with NAFLD.

Smoking is a major risk factor for chronic diseases, such as cardiovascular disease, diabetes, dyslipidemia, and hypertension, and has been reported to be associated with chronic liver disease and liver cancer [14–16]. In particular, smoking has been reported to be associated with NAFLD in several studies [17–21]. A meta-analysis reported a significant relationship between smoking and NAFLD [22]. However, several studies have reported that smoking and NAFLD are either unrelated or have only a weak relationship [23,24]. As such, the relationship between smoking and NAFLD remains controversial. Experimental studies have shown that nicotine alters the gut microbiota, increases intestinal permeability, and promotes hepatic lipid accumulation through oxidative stress and inflammatory signaling pathways [25,26]. These findings provide mechanistic support for the plausibility of smoking as a contributor to NAFLD pathogenesis.

Owing to the various health problems associated with smoking, interest in health management and smoking cessation is increasing worldwide. In previous studies investigating smoking cessation, many meaningful results related to the smoking cessation period have been reported. A recent study involving Korean male ex-smokers reported that the longer the smoking cessation period, the lower the insulin resistance [27]. However, few studies have investigated the association between smoking cessation and NAFLD.

Although smoking is a well-known modifiable risk factor for several chronic diseases, its role in the development of NAFLD remains incompletely understood. Notably, the impact of the duration of smoking cessation on NAFLD risk has been insufficiently explored, despite its practical relevance for public health strategies. Given that smoking behavior is a targetable lifestyle factor, elucidating the temporal association between cessation and NAFLD risk may offer valuable insight for preventive interventions. Therefore, this study aimed to investigate the association between smoking status—including cessation duration and cumulative exposure—and NAFLD in a nationally representative sample of Korean men.

## 2 Materials and methods

### 2.1 Subjects

Between January 2018 and December 2019, 19,919 subjects ≥ 20 years of age, who received medical checkups at the Health Examination Center of Soonchunhyang University Cheonan Hospital in Korea, were surveyed. Among females, the proportion of smokers was very low (1.26%).

We included adult male participants with complete information on anthropometric measurements, laboratory data, abdominal ultrasonography, and smoking history. Participants were excluded if they had missing physical measurement data (n = 506), missing smoking survey data (n = 597), missing blood test results (n = 628), or missing abdominal ultrasonography data (n = 494). We also excluded those with excessive alcohol consumption (≥ 210 g/week, n = 4,054), positive or missing results for hepatitis B surface antigen or hepatitis C antibody (n = 986), and those with a history or current treatment for cancer, chronic liver disease, cirrhosis, or hepatitis (n = 413).

After applying these criteria, 12,241 adult male participants were included in the final analysis.

### 2.2 Variables

The following information was obtained using a self-administered questionnaire: drug use; drinking history (number of alcoholic drinks per week; type of alcohol consumed [soju, beer, makgeolli, wine, and liquor]; and amount of alcohol consumed in one sitting); and smoking history. Alcohol consumption, medical history, and medication use were assessed using a self-administered health survey. The amount of alcohol consumed per week was calculated using the following equation [28].


Alcohol(g)=amountofalcohol(ml)×alcoholcontent(%)×alcoholspecificgravity(0.79)/100

Among those who had smoked > 100 cigarettes in their lifetime, those who smoked until recently were classified as current smokers; those who did not currently smoke were classified as ex-smokers; and those who did not smoke a single cigarette in their lifetime or smoked < 100 cigarettes in their lifetime were classified as never smokers [29]. Pack-years smoked were calculated by multiplying the number of packs smoked per day by the number of years smoking. Drinking was classified as > 10 g alcohol/day or < 10 g/day.

Body mass index (BMI) was calculated by dividing weight in kg (measured using an electronic body composition analyzer [X-scan plus 2, Jawon Medical Co. Ltd., Korea]) by height squared (m^2^). Obesity was defined as a BMI ≥ 25.0 kg/m^2^ according to the Asia-Pacific Obesity Treatment Guidelines [30].

Blood pressure was the mean of two measurements recorded from the upper left arm using an ESAY X 900 device (Jawon Medical CO., Ltd., Korea; Colin Electronics Co., Ltd., Aichi, Japan). For blood pressure measurements ≥ 140/90 mmHg, a manual blood pressure meter was used for reassessment and considered to be the final value.

After fasting for > 10 h, peripheral venous blood samples were collected for the assessment of total cholesterol (TC), triglycerides (TG), high-density lipoprotein cholesterol (HDL-c), low-density lipoprotein cholesterol (LDL-c), fasting blood glucose (FBG), and glycated hemoglobin (HbA1c).

Hypertension was defined as systolic blood pressure ≥ 140 mmHg, diastolic blood pressure ≥ 90 mmHg, or drug treatment for hypertension. Dyslipidemia was defined as TC ≥ 240 mg/dl, LDL-c ≥ 160 mg/dl, HDL-c < 40 mg/dl, TG ≥ 200 mg/dl, or drug treatment for dyslipidemia [31]. Diabetes was defined as FBG ≥ 126 mg/dl, HbA1c ≥ 6.5%, or taking a diabetes drug.

Abdominal ultrasonography was conducted using an EPIQ 5G device (Philips Healthcare, USA) to evaluate hepatic steatosis. The images were interpreted independently by two board-certified radiologists blinded to participants’ clinical data. Fatty liver was diagnosed when increased hepatic parenchymal echogenicity, posterior beam attenuation, and blurring of intrahepatic vessels were observed. The severity of hepatic steatosis was categorized as mild, moderate, or severe based on echogenicity, acoustic attenuation, and visualization of intrahepatic vessels and diaphragm [32]. In this study, any degree of steatosis classified as mild or greater was defined as NAFLD.

### 2.3 Statistical analysis

Subjects were categorized into 3 groups according to smoking status: non-smokers; ex-smokers; and current smokers. The Kruskal–Wallis test was used to compare continuous variables across smoking status groups because the data did not meet the assumption of normality. For post hoc pairwise comparisons after the Kruskal–Wallis test, the Mann–Whitney U test was used with Bonferroni correction. The Bonferroni-adjusted significance level was calculated by dividing 0.05 by the number of pairwise comparisons. Logistic regression analysis was employed to evaluate the association between smoking status and non-alcoholic fatty liver disease (NAFLD), yielding adjusted odds ratios (ORs) and corresponding 95% confidence intervals (CIs) as measures of association. Separate multivariable logistic regression models were conducted for each exposure variable (smoking status, cumulative pack-years, cessation duration), and subgroup analyses were performed where appropriate. To assess multicollinearity among covariates, we calculated variance inflation factors (VIFs) before performing multivariable logistic regression. All VIF values were below 2.0, indicating no significant multicollinearity. To assess potential information loss from categorization and examine nonlinear trends, smoking pack-years and cessation duration were also analyzed as continuous variables in supplementary models. Finally, smoking status was divided into never smoker, ex-smoker, and current smoker, and current smokers were divided according to smoking age, BMI, drinking, hypertension, dyslipidemia, and diabetes using the stepwise selection method (Table 2).

Ex-smokers were divided into 3 categories according to the length of the cessation: < 10 years; 10–20 years; and ≥ 20 years. Logistic regression analysis was performed to determine the association with NAFLD according to the smoking cessation period. Group variables divided according to the smoking cessation period were used as fixed variables, and variable selection was performed for age, BMI, drinking, hypertension, dyslipidemia, diabetes, and smoking years using the stepwise selection method (Table 4). Interaction terms between smoking cessation duration and metabolic factors (BMI, hypertension, diabetes, and dyslipidemia) were tested to evaluate potential effect modification.

All statistical analyses were performed using Rex version 3.6.0 (Rex Soft Inc., Seoul, Korea) and spreadsheet-based statistical analysis software (Excel, Microsoft, Corp., Redmond, WA, USA). Differences with *P* < .05 were considered to be statistically significant.

### 2.4 Ethics statement

This study was conducted in accordance with Declaration of Helsinki. Written informed consent was obtained from all participants at the time of their visit, and participants were informed that they could withdraw from the study at any time without consequences. The study protocol was reviewed and approved by the Institutional Review Board of Soonchunhyang University Cheonan Hospital (IRB No. SCHCA 2022-04-024, approved on April 27, 2022). The data access for research purpose was initiated on May 6, 2022, and all procedures, including data access, collection and analysis, were performed after this approval date in accordance with the Personal Information Protection Act of Korea.

## 3 Results

### 3.1 Characteristics of the study population

The mean age and BMI of the subjects was 43.0 years and 24.6 kg/m^2^, respectively. According to smoking status, 31.59%, 40.14%, and 28.27% were nonsmokers, ex-smokers, and current smokers, respectively.

The prevalence of obesity, hypertension, diabetes, and dyslipidemia was higher in the current and ex-smoker group than in the never smoker group (*P *< 0.001). The prevalence of fatty liver in the entire cohort was 58.57%, and distributed according to smoking status as follows: never smoker, 53.61%; ex-smoker 62.39%; and current smoker, 58.67% (*P* < 0.001) (Table 1).

**Table 1:** Characteristics and prevalence of NAFLD according to self-reported smoking status.

	Total	Never smoker	Ex-smoker	Current smoker	P-value^†^
	(N = 12241)	(N = 3867)	(N = 4914)	(N = 3460)	
**Age (year)** ^**^	43 (37, 49)	40 (35, 47)^a^	44 (39, 51)^b^	44 (39, 50)^b^	<0.001^*^
**≥40 years**	8061 (65.85%)	2071 (53.56%)	3518 (71.59%)	2472 (71.45%)	<0.001
**BMI (kg/m**^**2**^) ^**^	24.6 (22.8, 26.7)	24.4 (22.7, 26.4)^a^	24.9 (23.1, 26.8)^b^	24.6 (22.6, 26.6)^a^	<0.001^*^
**Obesity(≥25 kg/m**^**2**^)	5604 (45.78%)	1619 (41.87%)	2428 (49.41%)	1557 (45.00%)	<0.001
**Hypertension**	2429 (19.84%)	660 (17.07%)	1108 (22.55%)	661 (19.1%)	<0.001
**Diabetes mellitus**	1111 (9.08%)	242 (6.26%)	492 (10.01%)	377 (10.9%)	<0.001
**Dyslipidemia**	6135 (50.12%)	1702 (44.01%)	2583 (52.56%)	1850 (53.47%)	<0.001
**Alcohol intake (>10g/d)**	4770 (38.97%)	1087 (28.11%)	1970 (40.09%)	1713 (49.51%)	<0.001
**Fatty liver**	7169(58.57%)	2073(53.61%)	3066(62.39%)	2030(58.67%)	<0.001

Abbreviations: BMI, body mass index; WC, waist circumference.

p-value^†^ by Chi-squared test.

p-value* by Kurskal-Wallis test.

p-value** by Mann-Whitney U test and adjusted by Bonferroni correction.

^a, b^ indicate the same letters have non-significant difference between groups.

### 3.2 Relationship between self-reported smoking status and NAFLD

The unadjusted OR between smoking status and NAFLD was 1.436 (95% CI 1.318–1.564, *P* < 0.001) in the ex-smoker group compared with the never smoker group, 1.232 (95% CI 1.090–1.392), *P* < 0.001) in the current smoker group with 10–20 pack-years, and 1.358 (95% CI 1.186–1.554, *P* < 0.001) in the current smoker group with > 20 pack-years.

After adjusting for age, BMI, alcohol consumption, hypertension, diabetes, and dyslipidemia, the OR for smoking status and NAFLD was 1.190 (95% CI 1.071–1.322, *P* = 0.001) in the ex-smoker group compared with the non-smoking group, 1.289 (95% CI 1.107–1.500, *P* = 0.001) in the current smoker group with 10–20 pack-years, and 1.235 (95% CI 1.043–1.461, *P *= 0.014) in the current smoker group with ≥ 20 pack-years.

**Fig 1:**
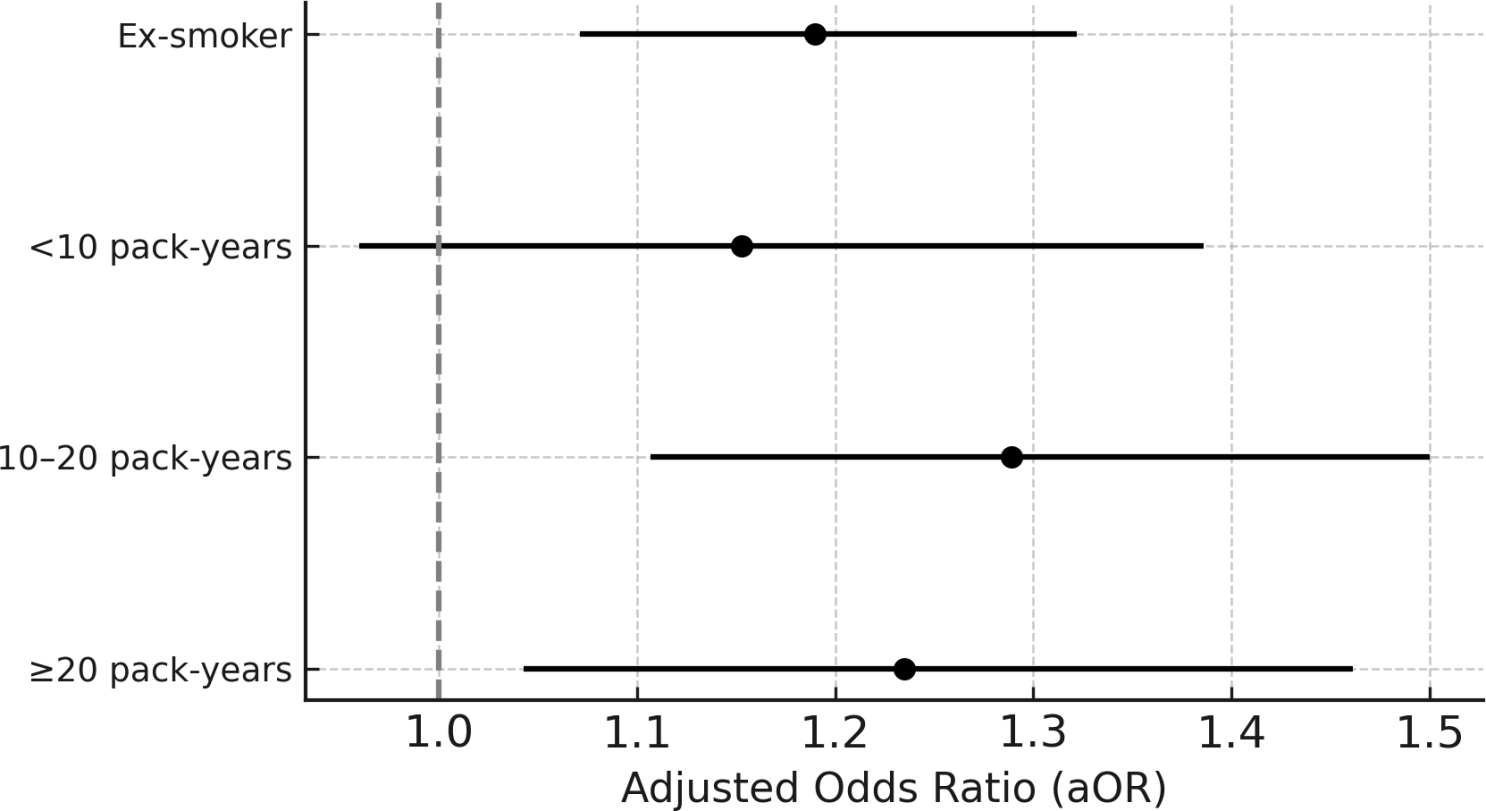
Adjusted odds ratios for NAFLD by pack-year. Adjusted odds ratios (aORs) and 95% confidence intervals for non-alcoholic fatty liver disease across categories of cumulative smoking exposure (pack-years). A higher cumulative exposure was associated with an increased risk of NAFLD. Analyses were adjusted for age, BMI, alcohol intake, hypertension, diabetes, and dyslipidemia.

Before and after adjusting for confounding variables, the OR was high in the ex-smoker and current smoker groups with > 10 pack-years, and the difference between the groups was statistically significant (Table 2 and Fig 1).

**Table 2:** Relationship of smoking and NAFLD.

	Unadjusted (95% CI)	P-value	Adjusted (95% CI)	P-value*
**Never**	1.000		1.000	
**Ex-smoker**	1.436 (1.318 - 1.564)	<0.001	1.190 (1.071 - 1.322)	0.001
**Current smoker**				
**<10 pack-years**	1.077 (0.930 - 1.248)	0.321	1.153 (0.960 - 1.386)	0.129
**10**–**20 pack-years**	1.232 (1.090 - 1.392)	<0.001	1.289 (1.107 - 1.500)	0.001
**≥20 pack-years**	1.358 (1.186 - 1.554)	<0.001	1.235 (1.043 - 1.461)	0.014

P-value* by adjusted for age, BMI, alcohol intake, hypertension, diabetes, dyslipidemia.

### 3.3 Characteristics of ex-smokers stratified according to duration of smoking cessation

Among smoking cessation periods, the proportion of smokers was distributed as follows: ex-smokers 45.54% < 10 years; 14.02% for 10–20 years, and 40.44% for ≥ 20 years. The mean age of the ex-smokers was 41.0 years in the group with a smoking cessation period < 10 years, 46.0 years in the group with 10–20 years, and 54.0 years in the group with ≥ 20 years, and the difference was statistically significant (*P* < 0.001).

BMI according to smoking cessation period was distributed as follows: < 10 years, 25.2 kg/m^2^; 10–20 years, 24.7 kg/m^2^; and ≥ 20 years, 24.6 kg/m^2^. There was a statistical difference between the groups with a smoking cessation period < 10 years versus > 10 years (*P* < 0.001).

The prevalence of NAFLD (defined as ≥ mild steatosis) was 65.08% in the group with a smoking cessation period < 10 years, 58.63% in the 10–20 years group, and 57.86% in the ≥ 20 years group (*P* < 0.001). There was a statistically significant difference between the groups (Table 3).

**Table 3:** Characteristics of ex-smokers stratified by the duration of smoking cessation.

	Duration of smoking cessation			P-value^†^
	< 10 years	10-20 years	> 20 years	
	(N = 2927)	(N = 1472)	(N = 515)	
**Age (year)** ^**^	41 (36, 47)^a^	46 (42, 53)^b^	54 (49, 59)^c^	<0.001^*^
**≥40 years**	1760 (60.13%)	1247 (84.71%)	511 (99.22%)	<0.001
**BMI (kg/m** ^2)**^	25.2 (23.3, 27.1)^a^	24.7 (23.1, 26.5)^b^	24.6 (22.8, 26.15)^b^	<0.001^*^
**Obesity(≥25 kg/m**^**2**^)	1538 (52.55%)	671 (45.58%)	219 (42.52%)	<0.001
**Alcohol intake (>10g/d)**	1220 (41.68%)	588 (39.95%)	162 (31.46%)	<0.001
**Hypertension**	556 (19%)	385 (26.15%)	167 (32.43%)	<0.001
**Diabetes mellitus**	286 (9.77%)	148 (10.05%)	58 (11.26%)	0.5814
**Dyslipidemia**	1566 (53.5%)	760 (51.63%)	257 (49.9%)	0.2221
**NAFLD**	1905 (65.08%)	863 (58.63%)	298 (57.86%)	<0.001

Data are presented as median (interquartile range) for continuous variables and n (%) for categorical variables.

Percentages for hepatic steatosis indicate the proportion within each cessation group.

Mild, moderate, and severe steatosis are collectively defined as NAFLD.

Abbreviations: BMI, body mass index; WC, waist circumference.

p-value† by Chi-squared test.

p-value* by Kurskal-Wallis test.

p-value** by Mann-Whitney U test and adjusted by Bonferroni correction.

^a, b, c^ indicate the same letters have non-significant difference between groups.

### 3.4 Relationship between smoking cessation and NAFLD

The unadjusted OR for NAFLD according to smoking cessation period in the group with cessation of 10–20 years was 0.760 (95% CI 0.669–0.865, *P* < 0.001) and 0.737 (95% CI 0.609–0.892, *P* < 0.001) in the group with cessation ≥ 20 years compared to those who had quit for < 10 years in the ex-smoker group.

**Fig 2:**
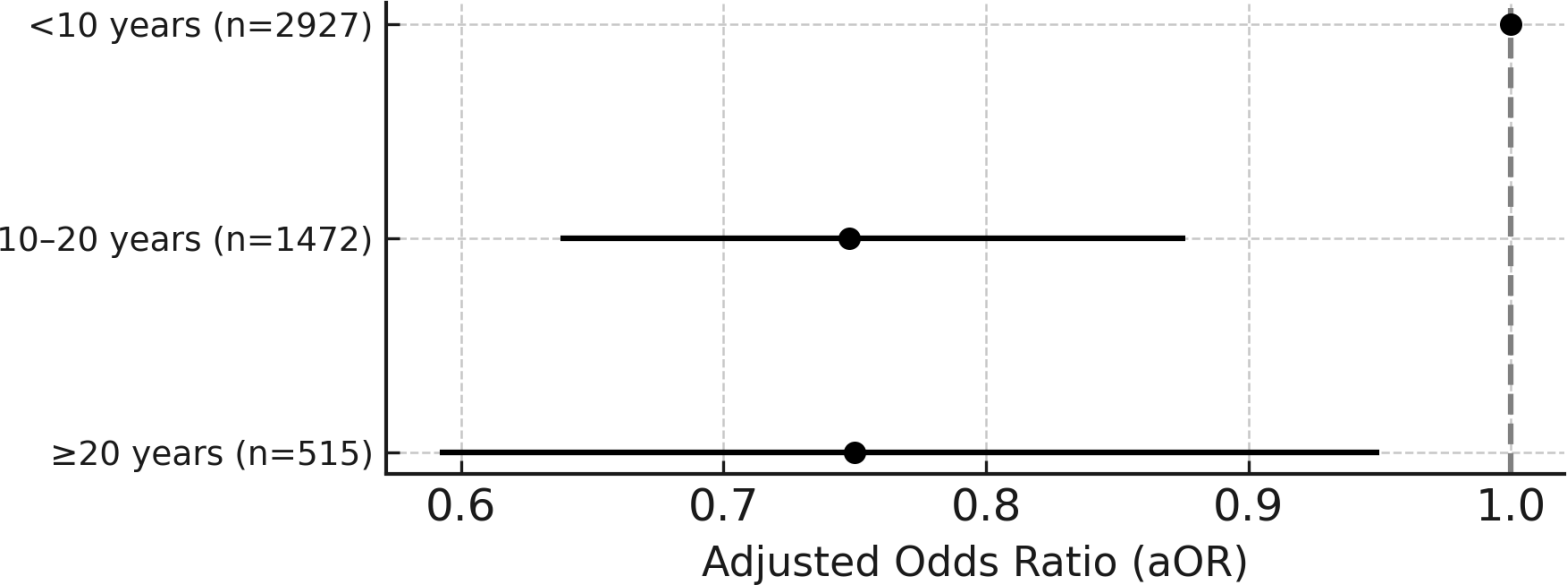
Adjusted odds ratios for NAFLD by smoking cessation duration. Adjusted odds ratios and 95% confidence intervals for NAFLD among ex-smokers, categorized by duration since cessation. Analyses were adjusted for age, BMI, alcohol intake, hypertension, diabetes, dyslipidemia, and cumulative pack-years. A significantly lower risk was observed in participants who had quit smoking for 10 years or more, compared to those with <10 years of cessation.

After adjusting for age, BMI, alcohol consumption, hypertension, diabetes, dyslipidemia, and pack-years smoked, the odds ratios (ORs) for NAFLD were 0.748 (95% CI: 0.638–0.876, *P* < 0.001) among those who had quit smoking for 10–20 years and 0.750 (95% CI: 0.592–0.950, *P *= 0.017) among those with cessation ≥ 20 years, compared to those who had quit for less than 10 years – a statistically significant difference (Table 4 and Fig 2).

**Table 4:** Relationship of smoking cessation and NAFLD.

	Unadjusted (95% CI)	P-value	Adjusted (95% CI)	P-value^*^
**Duration of smoking cessation**				
** < 10 years**	1.000		1.000	
** 10**–**20 years**	0.760 (0.669–0.865)	<0.001	0.748 (0.638–0.876)	<0.001
** > 20 years**	0.737 (0.609–0.892)	<0.001	0.750 (0.592–0.950)	0.017

P-value* by adjusted for age, BMI, alcohol intake, hypertension, diabetes, dyslipidemia, and pack-years.

When smoking pack-years and smoking cessation duration were analyzed as continuous variables, the results remained consistent with the grouped analysis. Greater cumulative smoking exposure was positively associated with the likelihood of NAFLD (OR 1.012, 95% CI 1.008–1.017, P < 0.001), whereas longer duration of smoking cessation demonstrated an inverse association (OR 0.985, 95% CI 0.976–0.993, P = 0.002).

## 4 Discussion

In this study, we investigated the relationship between NAFLD, smoking status, and pack-years of smoking among a sample of adult males, and the relationship with NAFLD after dividing ex-smokers according to the length of the smoking cessation period; specifically, < 10, 10–20, and ≥ 20 years.

The prevalence of NAFLD was 58.57% among all study participants, 53.61% in the never smoker group, 62.39% in the ex-smoker group, and 58.67% in the current smoker group. A meta-analysis examining the prevalence of NAFLD in Asia reported the prevalence of NAFLD in Korea diagnosed by abdominal ultrasound to be 32.9% [4]. The prevalence of NAFLD diagnosed by abdominal ultrasound in 141,610 medical examination recipients in Seoul and Gyeonggi Province was 34.4% among males [33].

The overall prevalence of NAFLD was higher than that in previous studies, and the characteristics of the study’s geographical area may have had an effect, which is believed to be related to the high prevalence of obesity among the subjects. According to national health statistics from 2019, the prevalence of obesity among adult males in Korea is 41.8%. Although it was not possible to determine weight change(s) of the subjects in this study, the prevalence of obesity was 49.41% in the ex-smoker group and 45.0% in the current smoker group, compared to 41.87%. In a cross-sectional study of Spanish adults aged 15–85 years, the prevalence of NAFLD was significantly higher among individuals with a BMI ≥ 25 kg/m^2^ compared to those with normal body weight [34].

In this study, there was a significant association between an increased risk for NAFLD in the ex-smoker group and the current smoker group that smoked > 10 pack-years. A study that confirmed smoking status using a self-administered questionnaire for 2691 Chinese males > 40 years of age reported a higher OR for NAFLD than never smokers in the ex-smokers and current smokers who smoked > 40 cigarettes per day [20]. This is also consistent with the results of a study that reported a significant relationship between pack-years of smoking and NAFLD in current smokers [35]. A recent large cohort study involving 199,468 Koreans reported a significant positive association between current smoking, pack-years smoked, urinary cotinine levels, and NAFLD [19]. In addition, a cross-sectional study using urinary cotinine levels as an objective indicator of smoking in 160,862 Koreans found that current smoking was associated with NAFLD [36].

However, other studies have reported contradictory results regarding the relationship between smoking and NAFLD. In a cross-sectional study involving 933 Mexican males and females, current smoking and pack-years smoked were not associated with NAFLD [23], although the number of study subjects was small, and the demographic and clinical characteristics of the smoking and non-smoking groups were similar.

Smoking was not related to NAFLD in a cross-sectional study that analyzed data from 11,003 Americans 20–74 years of age who participated in the Third National Health and Nutrition Examination Survey and evaluated smoking using a self-questionnaire and blood cotinine levels [37]. One possible explanation is that the effect of smoking on NAFLD may vary according to race.

The mechanisms underlying the effects of smoking on NAFLD remain unclear. Smoking has been reported to cause insulin resistance and is associated with increased levels of visceral fat [38,39]. NAFLD is associated with insulin resistance. This suggests that smoking negatively affects the pathophysiology of NAFLD. In particular, NAFLD is associated with an increased mortality rate due to cardiovascular diseases and complications of liver disease [40,41]. Therefore, it is important to identify subjects at high risk for developing NAFLD, to prevent its progression, and to design management and strategies to improve prognosis.

In this study, the risk for NAFLD increased among ex-smokers compared with never smokers; however, the risk for NAFLD decreased in those who quit for > 10 years compared to those who quit for < 10 years. Interestingly, in some subgroup comparisons, ex-smokers demonstrated slightly higher adjusted odds ratios for NAFLD than current smokers. This may be explained by residual hepatic effects of prior smoking, a lag in metabolic recovery after cessation, or health-related quitting bias, where individuals with emerging metabolic conditions are more likely to quit smoking, thereby which may enrich the ex-smoker group with higher baseline risk. A study of 13,466 Japanese individuals also reported that the risk for severely high fatty liver decreased in those who quit for > 10 years compared to those who quit for < 5 years [42]. In addition, a study of 851 Korean male ex-smokers reported that the longer the smoking cessation period, the lower the insulin resistance [27]. This finding suggests that smoking cessation can have a positive health effect on NAFLD. Although statistically significant, the observed effect sizes were relatively modest (aORs ~ 1.15–1.20). While these may have limited clinical impact on an individual level, they may still be relevant from a public health perspective, particularly given the high prevalence of NAFLD and the modifiable nature of smoking. Further research is required to determine whether NAFLD risk among smokers decreases with longer cessation. Additional research is required to consider the effects of weight gain, dietary changes, and physical activity.

The present study had several limitations, the first of which was its cross-sectional design, which precluded determination of the exact causal relationship between smoking and NAFLD. Second, the study participants attended a university hospital for medical examinations; thus, they are not representative of the general population. Third, abdominal ultrasonography was used instead of liver biopsy to diagnose NAFLD. Although abdominal ultrasound is not the most sensitive test for the diagnosis of NAFLD, it can diagnose fatty liver with high accuracy [43]. Additionally, liver biopsies are invasive and difficult to perform in large-scale epidemiological studies [44]. Therefore, many large-scale group studies have mainly used abdominal ultrasound to diagnose NAFLD [44,45]. Fourth, data regarding smoking were obtained only through a questionnaire, which may have introduced reporting bias caused by under-reporting of the actual amount of smoking. Fifth, confounding variables, such as daily physical activity, quality of diet, socioeconomic status, and regional characteristics, were not evaluated. As a result, residual confounding from these unmeasured variables may have influenced the observed associations and should be considered when interpreting the findings. In the future, more studies will be needed to clarify the effects of smoking on NAFLD.

## 5 Conclusion

In this study, the risk for NAFLD increased in adult males who quit smoking and smoked for > 10 pack-years, and decreased in those who quit smoking for < 10 years. Therefore, active smoking cessation management is necessary to prevent and treat NAFLD. In addition, studies that consider weight change, diet, and physical activity to characterize the pathophysiological association between smoking and NAFLD are warranted.
